# Supplementary material for: HP-Bodies – Ancestral Condensates that Regulate RNA Turnover and Protein Translation in Bacteria
Source: bioRxiv. 2025 Feb 6:2025.02.06.636932. Preprint. [Version 1] doi: 10.1101/2025.02.06.636932 (PMC11839049; doi:10.1101/2025.02.06.636932)
Supplement: Supplement 1 — Figure S1: PolyP-mediated Hfq focus formation in E. coli (related to Figure 1). Figure S2: Effects of polyP status on transcript stability in the N24 stress condition (related to Figure 5). Figure S3: Modulation of polyP levels in mammalian cells expressing yPPX or bacterial PPK (related to Figure 6). Table S1: Protein Composition of Hfq HMW complexes (related to Fig. 4). Table S2: GO-term enrichment analysis of HP body proteins (related to Fig. 4). Table S3: Strains and plasmids used in this study. [file NIHPP2025.02.06.636932v1-supplement-1.pdf]

bioRxiv preprint doi: https://doi.org/10.1101/2025.02.06.636932; this version posted February 6, 2025. The copyright holder for this preprint (which was not certified by peer review) is the author/funder, who has granted bioRxiv a license to display the preprint in perpetuity. It is made available under aCC-BY 4.0 International license.

Supplementary Dataset 1: Mass spectrometry results used for generating Table S1 and overlaps of GO terms annotated to proteins identified in the HP body vs. those in human P-bodies and stress granules (related to Fig. 4E). In the supplementary data file, the first tab gives more detailed descriptions of the data sets shown and the analysis used.

Supplementary Dataset 2: Fitted values and comparisons obtained from all high-throughput sequencing experiments used in this study. In the supplementary data file, the first tab gives more detailed descriptions of the data sets shown, the second tab gives the fitted values used in our analysis, and the third tab gives (specifically for the RNAseq datasets) q-values associated with each of the log fold changes given in the second tab.

bioRxiv preprint doi: https://doi.org/10.1101/2025.02.06.636932; this version posted February 6, 2025. The copyright holder for this preprint (which was not certified by peer review) is the author/funder, who has granted bioRxiv a license to display the preprint in perpetuity. It is made available under aCC-BY 4.0 International license.

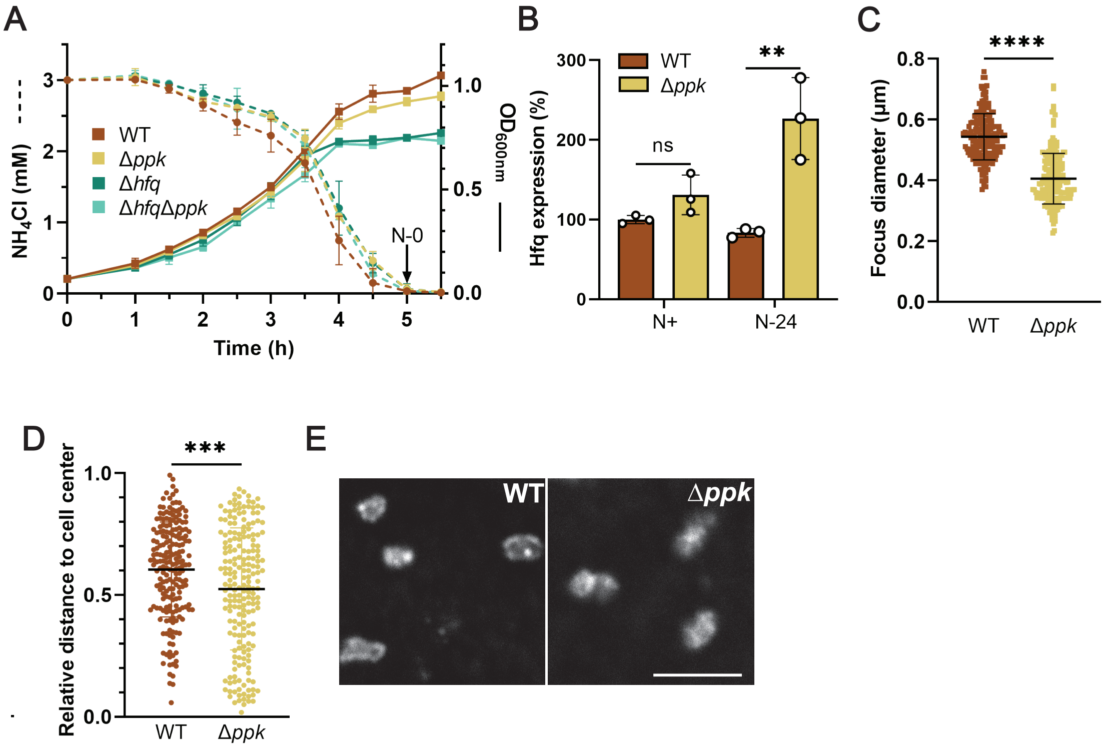

Figure S1: PolyP-mediated Hfq foc formation in *E. coli* (supplementing Figure 1)
(A) Growth (solid lines) and nitrogen (N) levels (dashed lines) of WT, Δ*ppk*, Δ*hfq* and Δ*hfq*Δ*ppk* *E. coli* MG1655 in Gutnick minimal medium supplemented with 3 mM NH_4Cl. Cells were grown at 37°C with aeration. Entry of N starvation (N0) is indicated. Error bars indicate SD (n=3). **(B)** Hfq-mCherry levels at N+ or N24 as determined by western blot using mCherry antibody. Total protein was used as loading control. Hfq-mCherry signal in N+ MG1655 *hfq::hfq-mCherry* cells was set to 100%. Error bars indicate SD (n=3). **(C)** Distribution of Hfq foci diameters in N24 in MG1655 *hfq::hfq-mCherry* WT and Δ*ppk* strains. Error bars indicate SD (n = 201). **(D)** Distribution of relative distances of Hfq condensates from 0 (cell center) to 1 (cell pole) in N24 in MG1655 *hfq::hfq-mCherry* WT and Δ*ppk* strains. Error bars indicate SD (n = 200). **(E)** Immunofluorescence images of fixed N24 WT or Δ*ppk E. coli* MG1655 *hfq::hfq-3xFLAG* using anti-FLAG antibodies for visualization. Comparisons in panels B-D were made by unpaired *t*-tests. ns, not significant; **p<0.01, ****p<0.0001, ****p<0.0001.

bioRxiv preprint doi: https://doi.org/10.1101/2025.02.06.636932; this version posted February 6, 2025. The copyright holder for this preprint (which was not certified by peer review) is the author/funder, who has granted bioRxiv a license to display the preprint in perpetuity. It is made available under aCC-BY 4.0 International license.

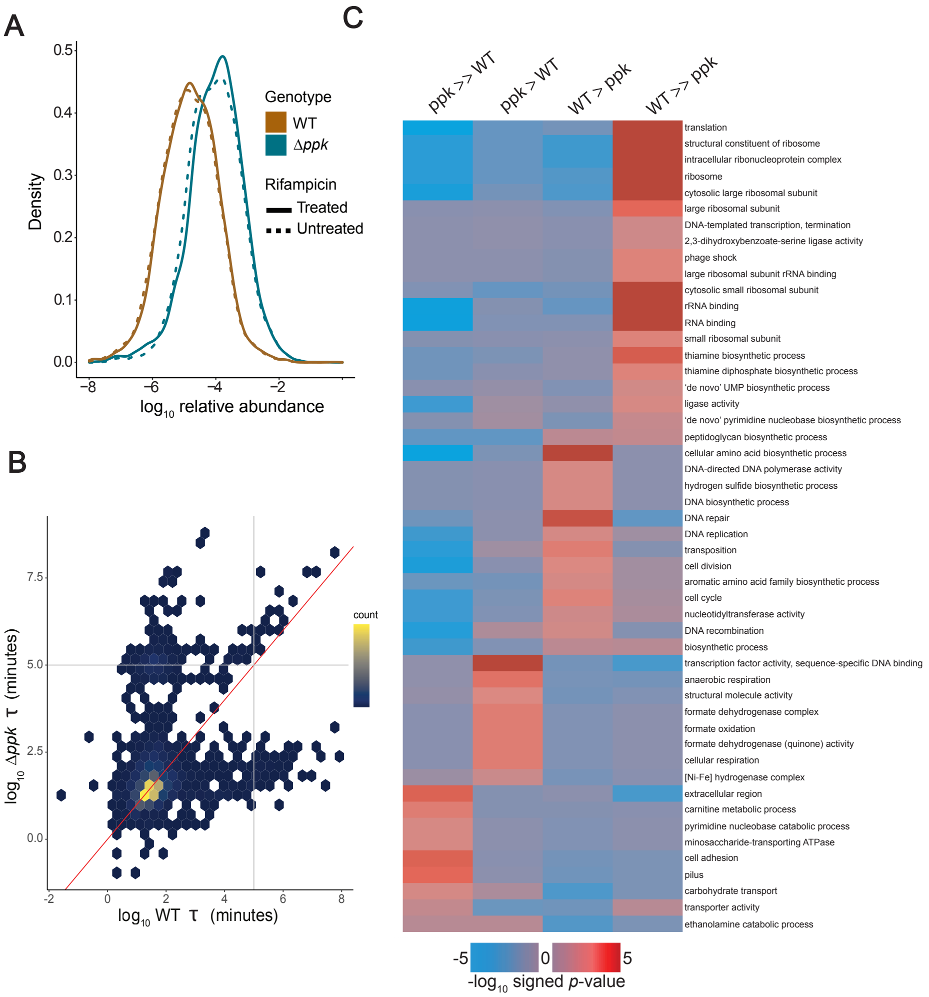

Figure S2. Effects of polyP status on transcript stability in the N24 stress condition (related to Figure 5). (**A**) Distributions of the observed transcript baseline abundances for the indicated combinations of genotype and rifampicin treatment. (**B**) Comparisons of fitted half-lives for transcripts in the WT vs *ppk* cell. (**C**) Full gene set enrichment analysis (performed using iPAGE software) on transcripts discretized into bins matching the categories in Fig. 5G.

bioRxiv preprint doi: https://doi.org/10.1101/2025.02.06.636932; this version posted February 6, 2025. The copyright holder for this preprint (which was not certified by peer review) is the author/funder, who has granted bioRxiv a license to display the preprint in perpetuity. It is made available under aCC-BY 4.0 International license.

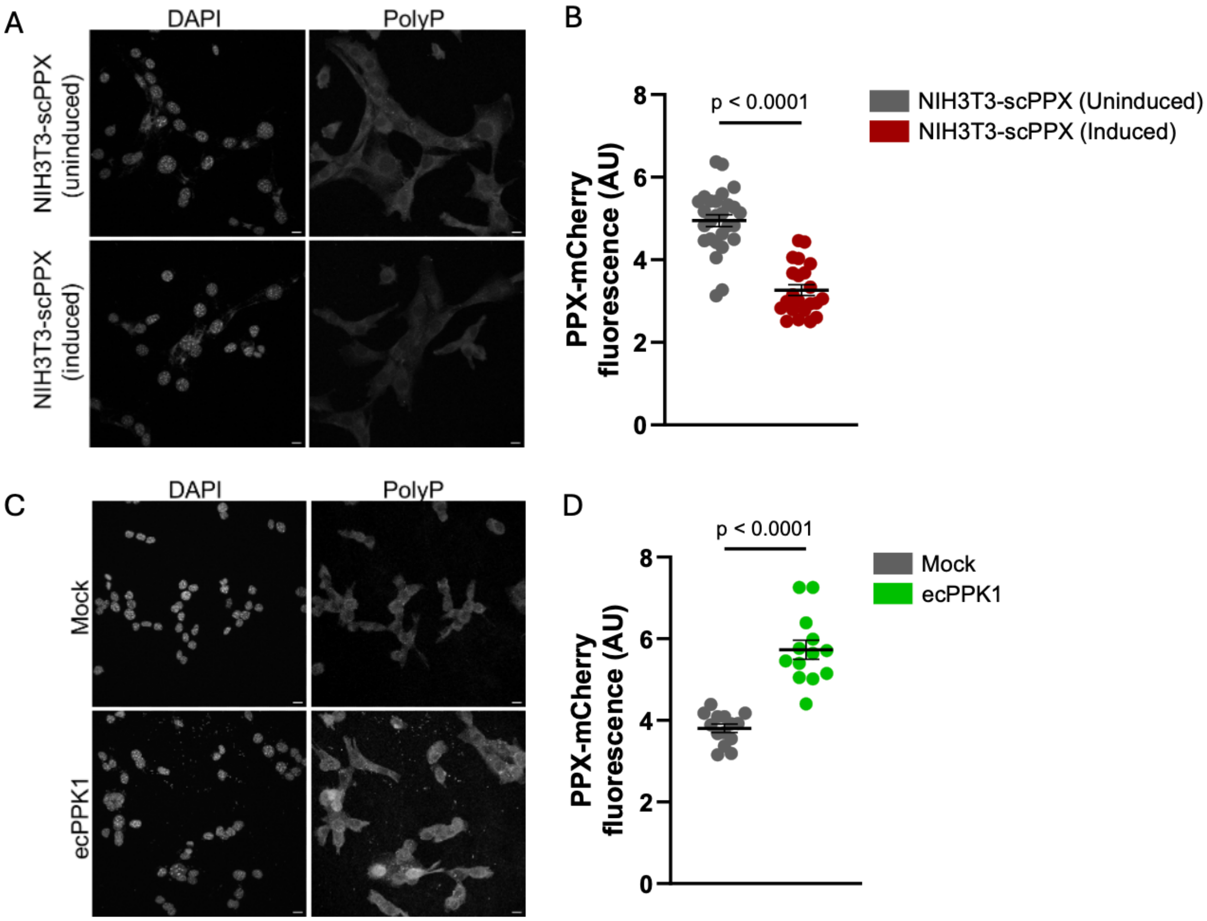

Figure S3: Manipulation of endogenous polyP levels in mammalian cells. (related to Figure 6). (A) A stable NIH3T3 cell line expressing a destabilization domain (DD) tagged yeast exopolyphosphatase (yPPX) was incubated in the absence (uninduced) and presence (induced) of small molecule, Shield1 (0.5 µM) that allows for the expression of yPPX. The cells were fixed and stained for polyP with PPXBD-mCherry. **(B)** PolyP levels were quantified based on fluorescence intensity of the PPXBD-mCherry probe. Each data point represents the average mCherry fluorescence intensity per cell (after background correction) in an image of at least 20 cells. **(C)** NIH3T3 cells were transiently transfected with a bacterial polyP kinase (ecPPK1) and were fixed and stained for polyP with PPXBD-mCherry. **(D)** PolyP levels were quantified based on fluorescence intensity of the PPXBD-mCherry probe. Each data point represents the mCherry fluorescence intensity of one cell (after background correction). An unpaired t-test was used to assess statistical significance (B, D). A representative set of images and quantification is shown (n=3). All scale bars are 10 µm.

bioRxiv preprint doi: https://doi.org/10.1101/2025.02.06.636932; this version posted February 6, 2025. The copyright holder for this preprint (which was not certified by peer review) is the author/funder, who has granted bioRxiv a license to display the preprint in perpetuity. It is made available under aCC-BY 4.0 International license.

Table S1: Proteins enriched in HP-HMW complexes (related to Figures 3, Supplementary data set 2). Lysates were prepared from N24 *hfq::hfq*-mCherry WT or the *hfq::hfq*-mCherry Δ*ppk* strain and analyzed on native PAGE. In addition, N24 *hfq::hfq*-mCherry WT lysate was treated with either yPPX to degrade polyP or RNaseA to degrade RNA prior to the native PAGE. Corresponding regions of the gels were excised, proteins pairwise differentially labelled (WT v *ppk*; WT v WT + yPPX; WT v WT + RNaseA) and analyzed by MS/MS analysis. Proteins with a log2 fold change (l2fc) >1 in at least 3 out of 5 replicates in WT v Δ*ppk*, 2 out of 4 replicates in WT v WT + yPPX or 2 out of 2 in WT v WT + RNase A are shown.

Acc #	Gene	Description	Enriched in WT lysate *versus*		
			**Δ*ppk***	**+ yPPX**	**+ RNaseA**
C4ZR49	**Hfq**	**RNA-binding protein**	**X**		
P0A7B1	Ppk	Polyphosphate kinase	**X**	**X**	
P0AG20	RelA	GTP pyrophosphokinase	**X**		
B1XAY9	HisS	Histidine--tRNA ligase	**X**	**X**	
B1XGY0	InfB	Translation initiation factor IF-2	**X**	**X**	**X**
C4ZYI0	InfC	Translation initiation factor IF-3	**X**	**X**	
C4ZZ48	RhlB	ATP-dependent RNA helicase	**X**	**X**	
P37765	RluB	Ribosomal large subunit pseudouridine synthase B	**X**	**X**	
P33643	RluD	Ribosomal large subunit pseudouridine synthase D	**X**	**X**	
C4ZQ93	RmlL	Ribosomal RNA large subunit methyltransferase I	**X**		
P21338	Rna	Ribonuclease I	**X**		
B1XDV3	RplL	50S ribosomal protein L9	**X**	**X**	
B1X6E6	RplQ	50S ribosomal protein L17	**X**	**X**	
C4ZUG4	RplX	50S ribosomal protein L24	**X**	**X**	
P0AG51	RpmD	50S ribosomal protein L30	**X**		
P0A7W1	RpsE	30S ribosomal protein S5	**X**	**X**	
P0ADZ4	RpsO	30S ribosomal protein S15	**X**		
P0A7T3	RpsP	30S ribosomal protein S16	**X**	**X**	
P0A7U3	RpsS	30S ribosomal protein S19	**X**	**X**	
P60390	RsmH	Ribosomal RNA small subunit methyltransferase H	**X**	**X**	
P14081	SelB	Selenocysteine-specific elongation factor	**X**		
B1XBU0	SmpB	SsrA-binding protein	**X**		
P00954	TrpS	Tryptophan--tRNA ligase	**X**	**X**	
B1XFU9	TyrS	Tyrosine--tRNA ligase	**X**	**X**	
P0AGJ5	YfiF	Uncharacterized tRNA/rRNA methyltransferase	**X**	**X**	
C4ZX86	Der	GTPase Der	**X**	**X**	
P0AEH5	ElaB	Protein ElaB	**X**		
B1XBZ0	RpoC	DNA-directed RNA polymerase subunit beta'	**X**		
B1X8V5	CbpA	Curved DNA-binding protein	**X**	**X**	
P0A972	CspE	Cold shock-like protein	**X**	**X**	
P0AES6	GyrB	DNA gyrase subunit B	**X**	**X**	
P0ACF0	HupA	DNA-binding protein HU-alpha	**X**	**X**	
P0ACF4	HupB	DNA-binding protein HU-beta	**X**	**X**	
C4ZQE5	RvuA	Holliday junction ATP-dependent DNA helicase	**X**	**X**	
P06612	TopA	DNA topoisomerase 1	**X**	**X**	
P0A698	UvrA	UvrABC system protein A	**X**	**X**	
P0A6Y8	DnaK	Chaperone protein DnaK	**X**		
P0A9M0	Lon	Lon protease	**X**	**X**	
P0AEU7	Skp	Chaperone protein Skp	**X**	**X**	
B1X927	AccD	Acetyl-coenzyme A carboxyl transferase sub B	**X**		
B1XDI9	Eno	Enolase	**X**	**X**	

bioRxiv preprint doi: https://doi.org/10.1101/2025.02.06.636932; this version posted February 6, 2025. The copyright holder for this preprint (which was not certified by peer review) is the author/funder, who has granted bioRxiv a license to display the preprint in perpetuity. It is made available under aCC-BY 4.0 International license.

P0A991	FbaB	Fructose-bisphosphate aldolase class 1	X	X	
P0A9B2	GapA	Glyceraldehyde-3-phosphate dehydrogenase A	X	X	X
P15877	Gcd	Quinoprotein glucose dehydrogenase	X	X	
P0A6V1	GlgC	Glucose-1-phosphate adenylyltransferase	X	X	X
P21599	PykA	Pyruvate kinase II	X	X	
P0AD61	PykF	Pyruvate kinase I	X	X	X
P0ABQ0	CoaBC	Coenzyme A biosynthesis bifunctional protein	X		
P38038	CysJ	Sulfite reductase [NADPH] flavoprotein alpha-comp	X	X	X
P0AA89	DosC	Diguanylate cyclase	X		
P0A6P7	EngB	Probable GTP-binding protein	X	X	
P39180	Flu	Antigen 43	X	X	
P63235	GadC	Glutamate/gamma-aminobutyrate antiporter	X	X	
P0ACC7	GlmU	Bifunctional protein	X	X	
P09831	GltB	Glutamate synthase [NADPH] large chain	X	X	X
P0ADG7	GuaB	Inosine-5'-monophosphate dehydrogenase	X	X	
P06987	HisB	Histidine biosynthesis bifunctional protein HisB	X		
C4ZXA5	IscS	Cysteine desulfurase IscS	X	X	
C4ZRS3	LpxA	Acyl- UDP-N-acetylglucosamine O-acyltransferase	X	X	
P21645	LpxD	UDP-3-O-(3-hydroxymyristoyl)glucosamine N-acyltransferase	X	X	
P45955	CpoB	Cell division coordinator	X	X	
P0AEZ3	MinD	Septum site-determining protein	X	X	
C4ZXV5	MoaC	Cyclic pyranopterin monophosphate synthase	X	X	
P02931	OmpF	Outer membrane porin F	X	X	
P76002	PliG	Inhibitor of g-type lysozyme	X	X	
P23865	Prc	Tail-specific protease	X	X	
P0AGC3	Slt	Soluble lytic murein transglycosylase	X	X	
C4ZRQ6	YaeH	UPF0325 protein	X	X	
P75863	YbcbX	Uncharacterized protein	X	X	
P0A9K3	YbeZ	PhoH-like protein	X	X	
P30177	YbiB	Uncharacterized protein	X	X	
P76177	YdgH	Protein YdgH	X	X	
P0ACY1	YdjA	Putative NAD(P)H nitroreductase	X	X	
P46853	YhhX	Uncharacterized oxidoreductase	X	X	
P76116	YncE	Uncharacterized protein YncE	X	X	

bioRxiv preprint doi: https://doi.org/10.1101/2025.02.06.636932; this version posted February 6, 2025. The copyright holder for this preprint (which was not certified by peer review) is the author/funder, who has granted bioRxiv a license to display the preprint in perpetuity. It is made available under aCC-BY 4.0 International license.

Table S2: GO-term enrichment analysis of HP body proteins (related to Fig, 4E). Shown are the set of GO terms present in the three-way interface between HP bodies, human P-bodies, and human stress granules.

DNA-related
GO:0003677 DNA binding
GO:0003697 single-stranded DNA binding
GO:0006281 DNA repair
GO:0006355 regulation of transcription, DNA-templated
RNA-related
GO:0003723 RNA binding
GO:0003724 RNA helicase activity
GO:0006401 RNA catabolic process
GO:0019843 rRNA binding
GO:0008298 intracellular mRNA localization
GO:1990904 ribonucleoprotein complex
GO:0008143 poly(A) binding
GO:0003676 nucleic acid binding
Translation related
GO:0003743 translation initiation factor activity
GO:0005840 ribosome
GO:0006412 translation
GO:0006417 regulation of translation
GO:0043022 ribosome binding
Stress related
GO:0009410 response to xenobiotic stimulus
GO:0009636 response to toxic substance
GO:0034605 cellular response to heat
GO:0042594 response to starvation
Locations
GO:0005576 extracellular region
GO:0005694 chromosome
GO:0005737 cytoplasm
GO:0005829 cytosol
GO:0016020 membrane
GO:0005886 plasma membrane
Other
GO:0000287 magnesium ion binding
GO:0005524 ATP binding
GO:0007165 signal transduction
GO:0008270 zinc ion binding

bioRxiv preprint doi: https://doi.org/10.1101/2025.02.06.636932; this version posted February 6, 2025. The copyright holder for this preprint (which was not certified by peer review) is the author/funder, who has granted bioRxiv a license to display the preprint in perpetuity. It is made available under aCC-BY 4.0 International license.

GO:0010468 regulation of gene expression
GO:0015031 protein transport
GO:0016301 kinase activity
GO:0016887 ATPase activity
GO:0042802 identical protein binding
GO:0042803 protein homodimerization activity
GO:0046872 metal ion binding
GO:0065003 protein-containing complex assembly
GO:0032991 protein-containing complex

Table S3. Strains, plasmids and cell lines used in this study.

**Bacterial strain**	**Marker (s)**	**Source**
MG1655 *(F-, λ-, rph-1 ilvG- rfb-50)*		(*1*)
MG1655 *Δppk*		(*2*)
MG1655 *Δhfq*		(*2*)
MG1655 *ΔhfqΔppk*		(*2*)
MG1655 *Δppx*		(*3*)
MG1655 *Δppk* pBAD18b	Amp	(*2*)
MG1655 *Δppk* pBAD18b *ppk*	Amp	(*2*)
MG1655 *Δppk* pBAD18b *hfq*	Amp	(*2*)
MG1655 *Δhfq* pBAD18b	Amp	(*2*)
MG1655 *Δhfq* pBAD18b *ppk*	Amp	(*2*)
MG1655 *Δhfq* pBAD18b *hfq*	Amp	(*2*)
MG1655 *hfq::hfq-PAmcherry*		(*2*)
MG1655 *hfq::hfq-PAmcherry Δppk*		(*2*)
MG1655 *hfq::hfq-mCherry*		This study
MG1655 *hfq::hfq-mcherry Δppk*		This study
MG1655 *hfq::hfq-mcherry Δppx*		This study
MG1655 *hfq::hfq*-3xFLAG		
MG1655 *hfq::hfq*-3xFLAG *Δppk*		
MG1655 *hfq::hfq-mCherry rne::rne-mTurquoise2*		This study
MG1655 *hfq::hfq-mCherry rhlB::rhlB-mTurquoise2*		This study
MG1655 *hfq::hfq-mCherry pnp::pnp-mTurquoise2*		This study
MG1655 *hfq::hfq-mCherry eno::eno-mTurquoise2*		This study
MG1655 *hfq::hfq-mCherry rne::rne-mTurquoise2 Δppk*		This study
MG1655 *hfq::hfq-mCherry rhlB::rhlB-mTurquoise2 Δppk*		This study
MG1655 *hfq::hfq-mCherry pnp::pnp-mTurquoise2 Δppk*		This study
MG1655 *hfq::hfq-mCherry eno::eno-mTurquoise2 Δppk*		This study
MG1655 *hfq::hfq-mCherry Δppk* pWSK129	Kan	This study
MG1655 *hfq::hfq-mCherry Δppk* pWSK129-*ppk*	Kan	This study
MG1655 *hfq::hfq-mCherry Δppk* pWSK129-*ppk10k*	Kan	This study
**Mammalian cell line**		

bioRxiv preprint doi: https://doi.org/10.1101/2025.02.06.636932; this version posted February 6, 2025. The copyright holder for this preprint (which was not certified by peer review) is the author/funder, who has granted bioRxiv a license to display the preprint in perpetuity. It is made available under aCC-BY 4.0 International license.

HEK293		ATCC® CRL-1573™	
HeLa		ATCC® CCL-2™	
NIH3T3		ATCC® CRL-1648™	
**Plasmid**	**Description**	**Markers**	**Source**
pKD4	Kanamycin resistance cassette donor	Kan	(*4*)
pKD46	λ red recombinase	Amp	(*4*)
pCP20	Flp recombinase	Amp	(*4*)
pWSK129	Low copy number vector	Kan	(*5*)
pWSK129 *ppk*		Kan	(*5*)
pWSK129 *ppk10k*		Kan	(*5*)
pcDNA3.1(-)	Mammalian expression vector	Amp	(*6*)
*Ec*PPK1	Mammalian expression vector for *E. coli* PPK	Amp	(*6*)
pLVX-pTuner Green	Mammalian expression vector for DD-tagged proteins	Amp	Takara
DD-3x-FLAG-scPPX	Mammalian expression vector for DD-tagged yeast PPX	Amp	This study
**Oligonucleotide**	**Sequence**		
JG-hfq-mFP-in-F	GCAACAGGACAGCGAAGAAACCGAATCGGCTGGCTCCGCTGCTGG		
JG-hfq-mFP-in-R	CCGTGTAAAAAAACAGCCCGAAACCCAAGCTTATGAATATCCTCCTTAGTTC		
JG-hfq-mFP-out-F	GTAGCAGCGCGCAGAATACTTCCGCGCAACAGGACAGCGAAGAAACCGAA		
JG-hfq-mFP-out-F	CGGGGAACGCAGGATCGCTGGCTCCCCGTGTAAAAAAACAGCCCGAAACC		
JG-rne-mFP-in-F	TCCTGCGCGTCCGCAACCTGTTGAGTCGGCTGGCTCCGCTGCTGG		
JG-rne-mFP-out-F	CGGCAACACATCATGCCTCTGCCGCTCCTGCGCGTCCGCAAC		
JG-rne-mFP-in-R	GGGCTTGATTACTTTGAGCTAATTACAAGCTTATGAATATCCTCCTTAGTTC		
JG-rne-mFP-out-R	AATAAAAAAGCCCTGGCAGTTACCAGGGCTTGATTACTTTGAGCTAATTAC		
JG-rhlb-mFP-in-F	TCCGCGTAATCGTCGTCGTTCAGGTTCGGCTGGCTCCGCTGCTGG		
JG-rhlb-mFP-in-R	TGATTTTGAGTATGACATTTTTTATCAAGCTTATGAATATCCTCCTTAGTTC		
JG-rhlb-mFP-out-F	GCAATGGTCCGCGTCGTACTGGCGCTCCGCGTAATCGTCGTCGTTC		
JG-rhlb-mFP-out-R	TCTTGCCATCTTGATACAGTTTGAATGATTTTGAGTATGACATTTTTTATCAAGC		
JG-pnp-mFP-in-F	AGCTCCGGCTGCTGAACAGGGCGAGTCGGCTGGCTCCGCTGCTGG		

bioRxiv preprint doi: https://doi.org/10.1101/2025.02.06.636932; this version posted February 6, 2025. The copyright holder for this preprint (which was not certified by peer review) is the author/funder, who has granted bioRxiv a license to display the preprint in perpetuity. It is made available under aCC-BY 4.0 International license.

JG-pnp-mFP-in-R	CCGCAGCGGAGGGCAAATGGCAACCCAAGCTTATGAATATCCTCCTTAGTTC
JG-pnp-mFP-out-F	AGTCTCAACCTGCTGCAGCACCGGAAGCTCCGGCTGCTGAACAG
JG-pnp-mFP-out-R	GTCCTGCCCGGTTAAAAGCCCCCCGCCGCAGCGGAGGGCAAATG
JG-eno-mFP-in-F	TCGTAAAGAGATCAAAGGCCAGGCATCGGCTGGCTCCGCTGCTGG
JG-eno-mFP-in-R	ATTTTTAAATCAGATAAAGTCAGTCCAAGCTTATGAATATCCTCCTTAGTTC
JG-eno-mFP-out-F	TGGGCGAAAAAGCACCGTACAACGGTCGTAAAGAGATCAAAGGCCAGG
JG-eno-mFP-out-R	AAAAAATGCCAGCCCGGAGGCTGGCATTTTTAAATCAGATAAAGTCAGTCCAAGC
JG-hfq-seq-F	ATGGCTAAGGGGCAATCTTTACAAG
JG-rne-seq-F	TTGAAACGGTAGCTGCGGTC
JG-rhlb-seq-F	ATTGGTCGTACAGGTCGCG
JG-pnp-seq-F	TGACTTTGGCGCATTTGTTGC
JG-eno-seq-F	CTGCAATCAAGATGGCGAAAGATG
JG-mFP-seq-R	GCCCAGTAGCTGACATTCATC

1. F. R. Blattner *et al.*, The complete genome sequence of Escherichia coli K-12. *Science* **277**, 1453-+ (1997).
2. F. Beaufay *et al.*, Polyphosphate drives bacterial heterochromatin formation. *Sci Adv* **7**, (2021).
3. M. J. Gray *et al.*, Polyphosphate Is a Primordial Chaperone. *Mol Cell* **53**, 689-699 (2014).
4. K. A. Datsenko, B. L. Wanner, One-step inactivation of chromosomal genes in Escherichia coli K-12 using PCR products. *P Natl Acad Sci USA* **97**, 6640-6645 (2000).
5. A. K. Rudat, A. Pokhrel, T. J. Green, M. J. Gray, Mutations in Escherichia coli Polyphosphate Kinase That Lead to Dramatically Increased In Vivo Polyphosphate Levels. *J Bacteriol* **200**, (2018).
6. A. Bentley-DeSousa *et al.*, A Screen for Candidate Targets of Lysine Polyphosphorylation Uncovers a Conserved Network Implicated in Ribosome Biogenesis. *Cell Reports* **22**, 3427-3439 (2018).
